# “Five Keys to Safer Food” and COVID-19

**DOI:** 10.3390/nu13124491

**Published:** 2021-12-15

**Authors:** Nadia San Onofre, Carla Soler, J. Francisco Merino-Torres, Jose M. Soriano

**Affiliations:** 1 Food & Health Lab, Institute of Materials Science, University of Valencia, Paterna, 46980 Valencia, Spain; sanonofre.nadia@gmail.com (N.S.O.); carla.soler@uv.es (C.S.); 2 Joint Research Unit on Endocrinology, Nutrition and Clinical Dietetics, University of Valencia-Health Research Institute La Fe, 46026 Valencia, Spain; merino_jfr@gva.es; 3 Department of Endocrinology and Nutrition, University and Polytechnic Hospital La Fe, 46026 Valencia, Spain

**Keywords:** COVID-19, food safety, “Five Keys to Safer Food” manual

## Abstract

On 11 March 2020, coronavirus disease 2019 (COVID-19) was declared a pandemic by the World Health Organization (WHO) and, up to 18:37 a.m. on 9 December 2021, it has produced 268,440,530 cases and 5,299,511 deaths. This disease, in some patients, included pneumonia and shortness of breath, being transmitted through droplets and aerosols. To date, there is no scientific literature to justify transmission directly from foods. In this review, we applied the precautionary principle for the home and the food industry using the known “Five Keys to Safer Food” manual developed by the World Health Organization (WHO) and extended punctually in its core information from five keys, in the light of new COVID-19 evidence, to guarantee a possible food safety tool.

## 1. Introduction

Coronavirus disease 2019 (COVID-19) is a viral pneumonia that was first identified in Wuhan (China) in December 2019 [1], which is caused by a β-coronavirus, like the severe acute respiratory syndrome coronavirus (SARS-CoV) [2] and the Middle East respiratory syndrome coronavirus (MERS-CoV) [3]. It was renamed by the Coronaviridae Study Group of the International Committee on Taxonomy of Viruses [4] as SARS-CoV-2 from 2019-nCoV and declared a pandemic by the World Health Organization [5] on March 11, 2020. Some patients′ common symptoms include fever and radiographic imaging reflecting pneumonia, which can develop from acute respiratory distress syndrome to death [6]. Nowadays, the appropriate indications of the WHO [7] about the introduction of physical distancing measures is useful and effective as one of the ways to reduce this disease. A range of 104 to 108 COVID-19 RNAs/g from stool samples has been isolated from infected patients [8,9,10], implying a risk of fecal-oral transmission [11] and prompting several authors to hypothesize transmission directly from foods [12,13,14]. However, to date, there is no literature regarding SARS-CoV-2 as a source of this transmission route [15] and consumers worldwide have been concerned about possible relationships with foods, affecting food imports from China [16]. In fact, Oakenfull and Wilson [17] indicated two exposure routes to SARS-CoV-2: (i) food intake (mainly eggs, meat, milk, dairy, and blood products) from contaminated animals, or (ii) intake of foodstuffs cross-contaminated by foods of animal and non-animal origin, used contact materials, or infected individuals who work in food preparation. Furthermore, Charlebois and Music [18] reflected that people were not prepared to work with food safety modifications as a result of SARS-CoV-2 in Canada. For all these reasons, the precautionary principle in the food safety chain must be a worldwide prioritization strategy. In recent years, the “Five Keys to Safer Food” [19] manual has been used as a key tool for food handlers to inform about responsibilities in the guarantee of food safety in industry and at home [19]. In our view, the WHO’s manual is more than ever a priority for use in the food industry and at home against COVID-19. This study was carried out with a special focus on the scientific development of COVID-19 and SARS-CoV-2 in relation to the five keys and their core information, to improve and guarantee food safety among the world′s population.

## 2. “Five Keys to Safer Food” Adapted to COVID-19

### 2.1. First Key: Keep Clean

#### 2.1.1. Wash Your Hands before Handling Food and Often during Food Preparation

Although, the prevalence of food handlers is currently considered very low [20], it is important to recommend regular handwashing before and after preparing or eating food [21]. The procedure of handwashing during food preparation should be recommended. Hand hygiene includes either cleaning hands with soap/water or with an alcohol-based hand rub, preferred if hands are or are not visibly dirty, respectively [22]. Ma et al. [23] studied several sanitizers that removed from 96.62 to 99.98% of the coronavirus from hands. These sanitizers must be applied to the skin, but never administered into the human body (through ingestion, injection, or any other route), which would result in poisoning people [24].

#### 2.1.2. Wash Your Hands after Going to the Toilet

The WHO [25] supported the efficacy of handwashing before eating and after using the toilet. In fact, this could be a source of contamination on the surfaces including by means of the ventilation system in a bathroom. In some cases, a review of a building’s pipes as transmission routes for patients with suspected COVID-19 infection should be raised by the Health Authorities to verify the safety of the building’s sewage system, as is reflected in a case [26] of two residents who lived on different floors of a building where a woman was diagnosed with COVID-19 about ten days after a man (aged 62 and 75 years, respectively), in the same building. Infected feces were spread through the building’s ventilation system, through the bathrooms, due to an improperly sealed tube.

#### 2.1.3. Wash and Sanitize All Surfaces and Equipment Used for Food Preparation

In food preparation in industry or at home there are commonly used surfaces and equipment made of several materials including stainless steel or plastic, among others. Van Doremalen et al. [27] estimated the decay rates of viable SARS-CoV-2 titers using Bayesian regression and observed that the estimated median half-life (t½) of SARS-CoV-2 was approximately 6.81, 5.63, 3.46, and 0.77 h on plastic, stainless steel, cardboard, and copper, respectively. However, Chin et al. [28] pipetted 5 μL droplets of virus culture (∼7 · 8 log unit of TCID50 per mL) on several surfaces, with a relative humidity of around 65% and at room temperature (22 °C), being immediately soaked with 200 μL of virus transport medium for 30 min to elute the virus. These authors indicated that this coronavirus was more stable on smooth surfaces and observed that no infectious virus could be detected after 3 h, and 2 and 4 days, for paper, wood, and glass, respectively, whereas no infectious virus could be detected for stainless steel and plastic on day 7. For food safety, a known concept is the presence on several surfaces of biofilms, mainly by bacteria, although viral biofilms can occur in them. In the host’s fluids, Thoulouze and Alcover [29] proposed that this viral biofilm is a risk of viral communities with enhanced infectious capacity and improved spread versus free viral particles, constituting a possible key reservoir for chronic infections. Andreu-Moreno and Sanjuán [30] defined to this community as “collective infectious units” (CIUs) and Tozzi, Peters, Annesi-Maesano and D’Amato [31] described this network-like appearance for SARS-CoV-2. However, although CIUs or biofilms for this coronavirus have not been reported on the surfaces and equipment used for food preparation, the cleaning process, including cleaning of all surfaces in the food location to remove biofilm, followed by proper disinfection, should be applied (Table 1). Chin et al. [28] added 15 μL of SARS-CoV-2 culture (∼7 · 8 log unit of TCID50 per mL) to 135 μL of several disinfectants at different working concentrations and observed that no infectious virus was detected after 5 min incubation at room temperature (22 °C) with benzalkonium chloride (0.1%), chlorhexidine (0.05%), chloroxylenol (0.05%), ethanol (70%), household bleach ((1:49) and (1:99)), and povidone-iodine (7.5%), while the use of hand soap solution (1:49) was useful after a 15 min incubation. Furthermore, this virus can be inactivated by UV [32] or heating at 56 °C for 30 min [33].

#### 2.1.4. Protect Kitchen Areas and Food from Insects, Pests, and Other Animals

Nowadays, there is no literature relating to SARS-CoV-2 transmitted by insects or pests, but Dehghani and Kassiri [34] suggested possible transmission by houseflies and cockroaches and recommended chemical (appropriate pesticides), physical (light and sticky traps, among others), and biological (use of parasitoids to control insects) methods in the food industry. However, bats [35] or probably pangolin species [36] could be natural reservoirs of SARS-CoV-2-like CoVs, but there is nothing to suggest these animals or others can contaminate kitchen areas.

### 2.2. Second Key: Separate Raw and Cooked

#### 2.2.1. Separate Raw Meat, Poultry, and Seafood from Other Foods

Although cross-contamination is a principal factor in food-borne illness outbreaks [37] including in Wuhan [38] and Hong Kong’s wet markets [39], the literature has not reflected this contamination implicating SARS-CoV-2, but the WHO [19] and Oakenfull and Wilson [17] indicated cross-contamination from raw meat, blood products, milk, or animal organs and suggested they should be handled with care; it should not be forgotten that two factors, intake of raw meat and humans who came into close contact with animals, have been suggested as the cause [40] for the initiation of COVID-19. The WHO [41] indicated that avoiding eating raw or undercooked animal products should be a general rule.

#### 2.2.2. Separate Raw Meat, Poultry, and Seafood from Other Foods

Equipment and utensils including cutting boards and knives should be separated when handling raw foods. Ouabdesselam and Sayad [42] and Khaniki and Salehi [43] reflected the importance of separating raw food from cooked food for the consumption of safe food during the COVID-19 pandemic.

#### 2.2.3. Store Food in Containers to Avoid Contact between Raw and Prepared Foods

There are two possible situations [44]; disposable or reusable containers. For the first, these materials are used in avoiding the need for cleaning of any returns, while the second involves implementation of appropriate sanitation and hygiene protocols. Their use is suggested to steer clear of contact from raw to prepared foods.

### 2.3. Third Key: Cook Thoroughly

#### 2.3.1. Cook Food Thoroughly, Especially Meat, Poultry, Eggs, and Seafood

The CDC [45], European Food Safety Authority [46], FDA [47], French Agency for Food, Environmental and Occupational Health & Safety [48], Institute of Food Technologists [49], USDA [50] and WHO [51], among others, have indicated that there is scientific scarcity to justify the food spread of COVID-19. However, several authors [52,53] indicated the possibility of fecal-oral transmission of SARS-CoV-2 due to the infectious agent scouring the gastrointestinal tract. In fact, Mycroft-West et al. [54] suggested that meat foods containing heparan sulphate (glycosaminoglycans) are highly charged anchors for this virus to interact with host tissue epithelia, and while this still needs to be studied as a source of possible foodborne transmission, this demonstrates that the guarantee of personal hygiene and sanitation protocols is absolutely associated with proper cooking to the right temperature [55]. In fact, meals that are prepared with raw meat products must be cooked with a core temperature around 70 °C, which must be achieved for a minimum of two minutes [56] or, according to the ANSES [48], heat treatment at 63 °C for four minutes to reduce contamination of SARS-CoV-2 in a food by a factor of 1000. These procedures will guarantee the right food procedure, with the assumption that ingested Coronavirus cannot survive the stomach acid [55].

#### 2.3.2. Bring Foods like Soups and Stews to Boiling to Make Sure That They Have Reached 70 °C. For Meat and Poultry, Make Sure That Juices Are Clear, Not Pink. Ideally, Use a Thermometer

Probable natural reservoirs of this virus are bats and pangolins, which are used to prepare soups in several countries [57]. For the first, soup of whole fruit bats, accompanied by coconut milk, vegetables and several spices, is cooked in Palauan restaurants [58], or soup of fruit bats, which have previously been washed and cooked, use all parts of this mammal such as fur, viscera, and wing membranes in Mariana Islands cuisine [59]. In fact, several authors [60,61,62] have speculated that consumption of soup of bats could be one of the probable causes of spreading of the infectious agent in China. For pangolin in Chinese cuisine, it is prepared in a hot pot [63] by mixing with other ingredients, as are caterpillar fungus (*Ophiocordyceps sinensis*) [64] or other meat including chicken or pork [65], among others. Independently of these mammals or other ingredients, any types of these dishes must be boiled to ensure a correct temperature, which should be verified with a thermometer.

#### 2.3.3. Reheat Cooked Food Thoroughly

Foods should reach a core temperature ≥72 °C for 2 min [66] or at least of 73.8 °C [67] on a food thermometer when safely reheated. Parvin et al. [68] indicated that a high temperature of at least 56 °C helps kill coronaviruses.

### 2.4. Fourth Key: Keep Clean

#### 2.4.1. Do Not Leave Cooked Food at Room Temperature for More Than 2 h

An instructor certified by the National Restaurant Association indicated the danger of exposing cooked food for too long at room temperature. [69].

#### 2.4.2. Refrigerate Promptly All Cooked and Perishable Food (Preferably below 5 °C)

An important warning is to refrigerate food for two hours after cooking and eat leftovers for up to three days or freeze for later use [70].

#### 2.4.3. Keep Cooked Food Piping Hot (More Than 60 °C) Prior to Serving

This core information is applied in this outbreak [71].

#### 2.4.4. Do Not Store Food too Long Even in the Refrigerator

Caution in relation to covered containers or sealed bags is also advised for storing food in the refrigerator [72].

#### 2.4.5. Do Not Thaw Frozen Food at Room Temperature

Three options could be selected: (i) defrosting food in the refrigerator, (ii) defrosting food in the microwave, and (iii) defrosting food as part of the cooking process [73].

### 2.5. Fifth Key: Use Safe Water and Raw Materials

#### 2.5.1. Use Safe Water or Treat It to Make It Safe

To date, the literature has not reflected the survival of this virus in drinking-water or sewage, while persistence in drinking-water is possible. However, there is a significantly high probability that its inactivation is faster than human enteric viruses (adenovirus, norovirus, rotavirus, and hepatitis A) [74].

#### 2.5.2. Select Fresh and Wholesome Foods

The importance of these is reflected in the COVID-19 measures in Costa Rica and Ireland, where fresh products, sourced from smallholder farmers for school feeding, are included in food baskets [75]. Furthermore, the use of alternative food networks based in family farming, along with conventional food networks, is useful in the prospective food shortages of this outbreak [76]. However, we must not forget the possible similarity of exposure to SARS-CoV-2 in these foods and the use of food disinfectants help to guarantee food safety for consumers. According to Table 1, some disinfectants are used in the food procedure. Ultraviolet is classified as UV-A, UV-B, and UV-C; the last, with wavelengths of 100 and 280 nm, is used for the disinfection of drinking water, wastewater, and sterilization of surfaces in contact with food, which favors DNA mutation. Kowalski et al. [77] proposed the use of 67 J/m^2^ as the mean D90 dose, which is the UV dose for 90% inactivation. However, the most widely used disinfectants for vegetables in restaurants were bleach and potassium permanganate (55 and 31%, respectively), followed by salt/lemon or soap (7% each) in the home [78,79].

#### 2.5.3. Select Fresh and Wholesome Foods

Shariatifar and Molaee-aghaee [80] recommended the boiling before use of raw and pasteurized milk.

#### 2.5.4. Do Not Use Food beyond Its Expiry Date

This information is even more important during this pandemic due to the tendency of the population to stockpile food regardless of its price, quality, and short expiration date. Long and Khoi [81] justified this situation, focusing on the fact that people consider that buying or not buying is safer, independent of the risks of high costs and even financial losses. Good management of these foods can be held either for the population or food industry, hoping that the term “accurate ordering” [82] is useful, which means taking account of expiry dates so as to minimize food wastage.

## 3. Conclusions

Nowadays, this pandemic is a global health crisis and the greatest worldwide challenge since World War Two. International scientific collaboration must be at all multidisciplinary, interdisciplinary, and transdisciplinary levels, and although there are no scientific findings to support SARS-CoV-2 being transmitted through foods, one strategy would be to apply the precautionary principle in the home and the food industry. In our view, the use of the “Five Keys to Safer Food” manual modified punctually with new scientific discoveries in connection with COVID-19 could be a good food safety tool due to the extensive use of the manual before this outbreak.

## Figures and Tables

**Table 1:** Inactivation of SARS-CoV-2 using different procedures.

Procedure	Time of Use	Performance Characteristics	Reference
Benzalkonium chloride (0.1%)	5 min	Virus titers were titrated using Vero-E6 cells in three independent triplicates taking into account that the detection limit of a typical 50% tissue culture infectious dose (TCID_50_) assay is 100 TCID_50_/mL, except reactions containing hand soap/chloroxylenol or reactions containing povidone-iodine/chlorhexidine/benzalkonium chloride with detection limits of 10^3^ and 10^4^ TCID_50_/mL, respectively, due to their cytotoxic effects.	[28]
Chlorhexidine (0.05%)	5 min		
Chloroxylenol (0.05%)	5 min		
Ethanol (70%)	5 min		
Household bleach (1:49)	5 min		
Household bleach (1:99)	5 min		
Povidone-iodine (7.5%)	5 min		
Soap solution (1:49)	15 min		
Ultraviolet	1 min 40 s	The assay was carried out as disinfection to deliver 1 J/cm^2^ for masks (distance from the UV lamp to masks) with an irradiance of 10 mW/cm^2^.	[32]
Heated at 56 °C	30 min	-	[33]
